# Virulence and Antimicrobial Resistance Patterns of *Salmonella* spp. Recovered From Migratory and Captive Wild Birds

**DOI:** 10.1002/vms3.70102

**Published:** 2024-11-04

**Authors:** Ruhena Begum, Nilima Akther Asha, Diponkar Chandra Chanda Dipu, Milton Roy, Asikur Rahman, Md. Shahidur Rahman Chowdhury, Hemayet Hossain, Md. Rafiqul Islam, Md Bashir Uddin, Md. Mahfujur Rahman, Md. Mukter Hossain

**Affiliations:** ^1^ Department of Medicine Sylhet Agricultural University Sylhet Bangladesh; ^2^ Department of Life Sciences Brunel University London, Kingston Lane Uxbridge Middlesex UK; ^3^ Department of Anatomy and Histology Sylhet Agricultural University Sylhet Bangladesh

**Keywords:** antimicrobial resistance, migratory birds, resistance gene, *Salmonella*, virulent gene, wild birds

## Abstract

**Background:**

*Salmonella* spp., especially those are resistant to extended‐spectrum β‐lactamase (ESBL), are considered as major concern to global health due to their emergence and dissemination.

**Aim:**

The aim of this study was to investigate the virulence and antimicrobial resistance (AMR) profile of *Salmonella* spp. from migratory and captive wild birds.

**Method:**

A total 262 faecal samples were collected, and the identification of *Salmonella* spp. was carried out using a standard culture and PCR as well as molecular detection of virulence and AMR genes.

**Results:**

The overall prevalence of *Salmonella* was determined to be 30.92% (95% CI = 25.63–36.75). Migratory birds exhibited highest prevalence (38.10%), whereas wild birds in captivity showed a lower prevalence (23.40%). The *agfA* gene was detected at a higher rate at 24.69%. *Salmonella* spp. exhibited 100% resistance to tetracycline, followed by 58% ampicillin and 46% streptomycin. In addition, there was a resistance rate to ceftriaxone of 17% and to colistin sulphate of 25%. Interestingly, levofloxacin alone displayed 100% sensitivity across all isolates, while ciprofloxacin and azithromycin showed 73% and 64% sensitivity, respectively. The MAR index was 0.25 and 0.42, and 74.07% of all isolates showed multidrug resistance (MDR). It was shown that migratory and captive wild birds contained ESBL genes *blaTEM* (94.34% and 49.06%) and *blaSHV* (13.33% and 10%), respectively. Genes responsible for sulphonamide (*sul1*) resistance were detected in 13.33% and 79% of wild and migratory birds, respectively.

**Conclusion:**

*Salmonella* has been found in captive wild and migratory birds and could act as reservoirs for the transmission of MDR and ESBL bacteria.

## Graphical abstract

Faecal samples were collected from migratory and wild birds. The prevalence of *Salmonella* was determined to be 30.92%. Migratory birds exhibited higher prevalence (38.10%) than wild birds (23.40%). The *agfA* gene was detected at a higher rate at 24.69%. *Salmonella* spp. exhibited 100% resistance to tetracycline whereas levofloxacin alone displays 100% sensitivity across all isolates, while ciprofloxacin and azithromycin showed 73% and 64% sensitivity, respectively. Migratory and captive wild birds contained ESBL genes *blaTEM* (94.34% and 49.06%) and *blaSHV* (13.33% and 10%), respectively.

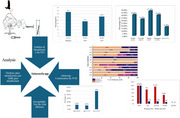

## 1 Introduction

The utilisation of antibiotics has witnessed a consistent rise since its initial application in the treatment of bacterial infections. Although antibiotics were initially developed to treat humans, their application has now extended to veterinary care and cattle production. The emergence of antimicrobial resistance (AMR), commonly referred to as multidrug resistance (MDR), can be attributed to the indiscriminate and haphazard utilisation of antibiotics across a multitude of bacterial strains in diverse environments. Consequently, the proliferation of antibiotic‐resistant bacteria manifests rapid dissemination from one environment to another, thereby disrupting the delicate equilibrium of global ecosystems. The propagation of resistant bacteria across various ecological elements is intrinsically intertwined with the improper administration of antibiotics usage and the presence of inadequate healthcare systems (Rashid, Rakib, and Hasan 2015). It is crucial to acknowledge that the prevalence of resistant microorganisms within the environment is directly regulated by the extent of antibiotic usage (Ramey et al. 2018). Intriguingly, even in untainted settings devoid of any direct association with human exposure, there exists a contributory link to the development of bacterial antibiotic resistance (Rashid, Rakib, and Hasan 2015). These results imply that additional elements are involved in the spread of resistant bacteria to these favourable environments. Both wild birds and migratory birds have the potential to serve as carriers and reservoirs of antimicrobial resistant microorganisms, which have significant implications for the health of both humans and animals (Saiful Islam et al. 2021).

In quest of the ideal biological settings and habitats suitable for breeding, nesting and raising their young, migratory birds traverse thousands of kilometres. Winters in Bangladesh are milder than those in the north because of its location in a subtropical area endowed with several transboundary rivers. Every year, a significant number of birds migrate to Bangladesh, driven by the country's favourable climatic conditions and abundant water resources, such as ponds, lakes and rivers. It is well recognised that migratory birds have a role in the transmission and spread of human and animal infections, encompassing bacteria, viruses, fungi, archaea and parasites. These birds can serve as either asymptomatic carriers or hosts for infected vectors (Benskin et al. 2009; Gogu‐Bogdan et al. 2014). According to several studies, migrating birds can be involved in the epidemiology of human and livestock salmonellosis due to their patterns of transmission of bacterial infections to aquatic environments (Hird et al. 2015; Kozak et al. 2013). Importantly, ducks and duck‐like birds can spread bacterial diseases to people, pets and livestock through water sources contaminated by the faeces of migratory birds (Akter et al. 2020). The function of wild birds as disease vectors may be underestimated, as many individuals may carry sub‐lethal amounts of potentially pathogenic bacterial species asymptomatically. However, recent research has revealed that many of the bacterial pathogens that affect poultry have also been identified in wild birds (Benskin et al. 2009). Due to a general lack of interest in wild birds as zoonotic vectors of disease combined with their low commercial value, few studies have examined their gastrointestinal flora (Kobuszewska and Wysok 2024). In places like eco parks, zoos and safari parks, among others, many wild birds are confined to cages. As a result of stress and immunosuppression, they frequently experience a variety of bacterial infections that frequently involve either natural flora or environmental pathogens (Fischer and Romero 2019).

In recent decades, zoonotic enteropathogenesis, including *Campylobacter* spp.*, Salmonella* spp. and *Vibrio* spp., as well as commensal pathogens, such as *Enterococcus* spp. and *Escherichia coli*, has developed AMR (Díaz‐Sánchez et al. 2012). Resistant *Salmonella* spp., a significant zoonotic pathogen, can result in enteric fever, gastroenteritis and even life‐threatening repercussions in humans as well as salmonellosis in poultry (Tawyabur et al. 2020). The *Salmonella* spp. spread to humans is predominantly influenced by the food chain. One of the known causes of death in wild and migratory birds is salmonellosis, which is instigated by *Salmonella* spp., notwithstanding the fact that seemingly healthy birds can also serve as carriers of these bacteria (Najdenski et al. 2018).

So far, very little information has been collected on the identification of the virulence genes and MDR *Salmonella* spp. in migratory and captive wild birds of the Sylhet district, Bangladesh. Due to their notable significance for public health, we have concentrated on the AMR profiles of *Salmonella* spp. in this investigation. The purpose of the current study was to enhance comprehension regarding the prevalence and AMR patterns, both phenotypically and genotypically, of *Salmonella* spp. in migratory and captive wild birds in the Sylhet region of Bangladesh.

## 2 Materials and Methods

### 2.1 Sample Collection and Processing

A cross‐sectional survey was conducted from January to July 2022 to assess the presence of migrating birds in Haripur, Bondor Bazaar and Sunamganj (Tanguar Haors). Furthermore, samples of captive wild birds were collected from the Bangladesh Bannyaprani Sheba Foundation (Zoo) in Srimongol and the Tilagor Eco Park in Sylhet. The study population comprised of a total 262 faecal and cloacal samples, with 128 samples from captive wild birds and 134 samples from migratory wild birds that were captured humanely for the purpose of sampling. Only fresh, moist faecal samples from captive wild birds were collected from the ground and foliage soon after defecation. This approach ensured that the samples belonged to the correct species and minimised environmental contamination. Samples from migratory wilds birds were collected directly from the cloaca using cotton swabs. The sterile conditions were maintained during sample collection. The samples were collected in accordance with the procedure outlined in a previous study (Akter et al. 2020). In brief, the droppings of the migratory birds and cloacal swabs of captive birds were carefully collected using a sterilised cotton bud. Each sample was then placed in an individual sterilised zip lock bag, labelled with a unique identification number and promptly (within 12 h) transported to the laboratory while ensuring cool chain using ice box.

### 2.2 Isolation and Identification of *Salmonella*

The isolation and identification of *Salmonella* were carried out according to the procedures outlined in the International Organization for Standardization (ISO 6579: 2002; amended version: ISO 6579‐1:2017/A1:2021) manual. The samples were subjected to the pre‐enrichment step by culturing them in buffered peptone water medium in a 1:10 ratio, and the samples were incubated at 37°C for 24 h. For *Salmonella*‐specific pre‐enrichment, cultures were further transferred 100 µL to modified semi‐solid Rappaport‐Vassiliadis (MSRV) agar (HiMedia, India) at three different spots and incubated at 42°C for 24 h. Following enrichment, a loopful of enriched broth was positive sample initially streaked on xylose‐lysine–deoxycholate (XLD) agar (HiMedia, India) and *Salmonella–Shigella* (SS) agar, and a single (pinkish with black centre) colony was randomly selected from each culture to continue with the identification. Colonies were identified as *Salmonella* based on their morphological and biochemical properties and Gram stain. To obtain greater reliability and validation in the process, two to three colonies from each monoclonal culture were tested biochemically by dilution streaking and stabbed onto triple sugar iron (TSI) agar, motility indole urea (MIU) and methyl red (MR), and tubes were incubated at 37°C for 16–24 h. Positive samples were further cultured on nutrient agar (NA) (Hi media, India) and incubated at a temperature of 37°C for 16–24 h, and pure isolates were stored in brain heart infusion broth (BHI) (Hi media, India) with 15% glycerol at −20°C for further use.

### 2.3 DNA Extraction

DNA from a pure culture was extracted using the conventional boiling method. Briefly, each isolate was cultured on NA and incubated overnight at 37°C. Following this, three to five fresh single colonies were harvested from the overnight culture and suspended in nuclease‐free water. The bacterial suspension was then boiled at 99°C for 10 min and subsequently cooled on ice for a period of 10 min. Finally, the debris was separated by centrifugation, and the supernatant was utilised as the DNA template for PCR (Khan et al. 2021; Uddin et al. 2021).

### 2.4 Molecular Detection of *Salmonella* and Virulence Genes

*Salmonella* was confirmed by the molecular detection of targeting *invA* gene using specific primers as previously described (Khan et al. 2021). The PCR assay was performed in the thermal cycler TC1000G PCR System (DLAB Scientific Inc., USA) with a heated lid. The cycling conditions included 50°C for 3 min (UDG reaction), 95°C for 10 min (initial denaturation), 35 cycles of 95°C for 30 s (denaturation), 68°C for 45 s (annealing) and 72°C for 5 min for final extension. All reaction mixtures, including the negative control and *Salmonella* positive DNA, were tested in duplicate in the same run of PCR assay. PCR products were analysed on 1.8% agarose gels stained with RedSafe^TM^ (iNtRON Biotechnology, Korea) nucleic acid staining solution (20,000×), photographed and stored as a digital image. All *Salmonella* isolates were screened for the presence of virulence genes by PCR. The PCRs were executed in single reactions employing specific primers (Table 1) for the detection of three virulence genes, namely *invA, agfA* and *sopE*. All PCR assays were optimised in a 25 µL reaction mixture containing 2 µL of DNA template, 12.5 µL of 2× master mix (Add Bio Inc., South Korea), 1 µL each of forward and reverse primers (10 pmol/µL) and 8.5 µL nuclease‐free water. The cycling conditions for the detection of *invA, agfA* and *sopE* genes were performed as previously described (Khan et al. 2021; Naidoo et al. 2022; Prager et al. 2003). The PCR products were analysed as described above.

**TABLE 1:** Primer sequences used in this study for the detection of virulence and resistance genes.

Target genes	Primer sequence (5′–3′)	Amplicon size (bp)	Annealing temperature	References
*invA*	TAATGCCAGACGAAAGAGCGT GATA TTGGTGTTTATGGGGTCGTT	100	68°C	Khan et al. (2021)
*agfA*	F: TCCACAATGGGGCGGCGGCG	350	50°C	Naidoo et al. (2022)
	R: CCTGACGCACCATTACGCTG			
*sopE*	F: GGATGCCTTCTGATGTTGACTGG	398	50°C	Naidoo et al. (2022), Prager et al. (2003)
	R: ACACACTTTCACCGAGGAAGCG			
*Sul1*	F: CGGCGTGGGCTACCTGAACG	433	66°C	Kozak et al. (2009)
	R: GCCGATCGCGTGAAGTTCCG			
*tet(A)*	F: GGCGGTCTTCTTCATCATGC	502	63°C	Kozak et al. (2009)
	R: CGGCAGGCAGAGCAAGTAGA			
*blaTEM*	F: CATTTCCGTGTCGCCCTTATTC	800	60°C	Dallenne et al. (2010), Islam et al. (2022)
	R: CGTTCATCCATAGTTGCCTGAC			
*blaSHV*	F: AGCCGCTTGAGCAAATTAAAC	713	60°C	Dallenne et al. (2010)
	R: ATCCCGCAGATAAATCACCAC			
*blaOXA*	R: ATCCCGCAGATAAATCACCAC	564	60°C	Dallenne et al. (2010)
	R: GACCCCAAGTTTCCTGTAAGTG			

### 2.5 Antimicrobial Susceptibility Test

Antimicrobial susceptibility testing (AST) was conducted using the Kirby–Bauer disk diffusion method in accordance with the guidelines of the Clinical and Laboratory Standards Institute (Humphries et al. 2022). The diameter of the zone of inhibition surrounding the disks was measured manually using millimetre scale and compared to the CLSI break points (CLSI 2022). Disk diffusion was carried out against 12 antimicrobials in 9 groups, including aminoglycosides: gentamicin (CN, 10 µg) and streptomycin (S, 10 µg); cephalosporin/β‐lactam antibiotics: ceftriaxone (CRO, 30 µg) and cefixime (CFM, 10 µg); β‐lactamase inhibitors: amoxicillin–clavulanate (AMC, 30 µg); penicillins: ampicillin (AMP, 10 µg); macrolides: azithromycin (AZM, 15 µg); quinolones/fluoroquinolones: ciprofloxacin (CIP, 5 µg) and levofloxacin (LEV, 5 µg); folate pathway inhibitors: sulphamethoxazole‐trimethoprim (SXT, 25 µg); tetracycline: tetracycline (TE, 10 µg); and polymyxins: colistin sulphate (CT, 10 µg). The manufacturer's guidelines from Oxoid, UK, were used to interpret the disk diffusion tests with colistin, as CLSI does not provide specific recommendations for colistin (Singh et al. 2022). The colistin sulphate cut value was recorded according to the manufacturer's guidelines. *Salmonella* isolates resistant to three or more antimicrobials were defined as MDR isolates (Magiorakos et al. 2012). The multiple antibiotic resistance (MAR) index was calculated and interpreted using the proven method (Adzitey, Rusul, and Huda 2012; Titilawo et al. 2015).

### 2.6 Molecular Detection of Resistance Genes

The detection of β‐lactamase genes (*blaTEM, blaSHV and blaOXA*), tetracycline (*tetA*) and genes resistant to sulphonamide (*sul1*) was performed by PCR using specific primers (Dallenne et al. 2010; Islam et al. 2022; Kozak et al. 2009) as listed in Table 1. All PCR assays were adjusted in a 25 µL reaction mixture containing 2 µL of DNA template, 12.5 µL of 2× master mix (Add Bio Inc., South Korea), 1 µL each of forward and reverse primers (10 pmol/µL) and 8.5 µL of nuclease‐free water. For the β‐lactam gene, multiplex PCR (m‐PCR I) amplification was carried out with an initial denaturation at 94°C for 10 min; 30 cycles of 94°C for 40 s, 60°C for 40 s and 72°C for 1 min; and a final elongation step at 72°C for 7 min. PCR test was conducted to detect sulphonamide‐resistant genes with an initial denaturation at 95°C for 15 min; 30 cycles of 95°C for 1 min, 1 min of annealing at 66°C and 72°C for 1 min; and a final elongation step at 72°C for 10 min. Similarly, PCR was performed to detect the resistance gene to tetracycline with an initial denaturation at 94°C for 15 min; 30 cycles of 94°C for 1 min, 1 min of annealing at 63°C and 72°C for 1 min; and a final elongation step at 72°C for 10 min. Amplicons were visualised after running at 100 V with 500 mA for 30 min on a 1.5% agarose gel containing Red SafeTM nucleic acid staining solution. A 100‐bp DNA ladder (Thermo Scientific, USA) was used as a size marker.

### 2.7 Statistical Analysis

Data obtained from the present study were inserted into Microsoft Excel 2013 (Los Angeles, CA, USA) and analysed by SPSS 26.0 (IBM SPSS Statistics version 26.0; IBM SPSS‐25.0, USA). A probability (*p*) value < 0.05 was considered as statistically significant. The precision of these estimates was ensured by calculating a 95% confidence interval for the proportions.

### 2.8 Plot and Geospatial Mapping

**FIGURE 1:**
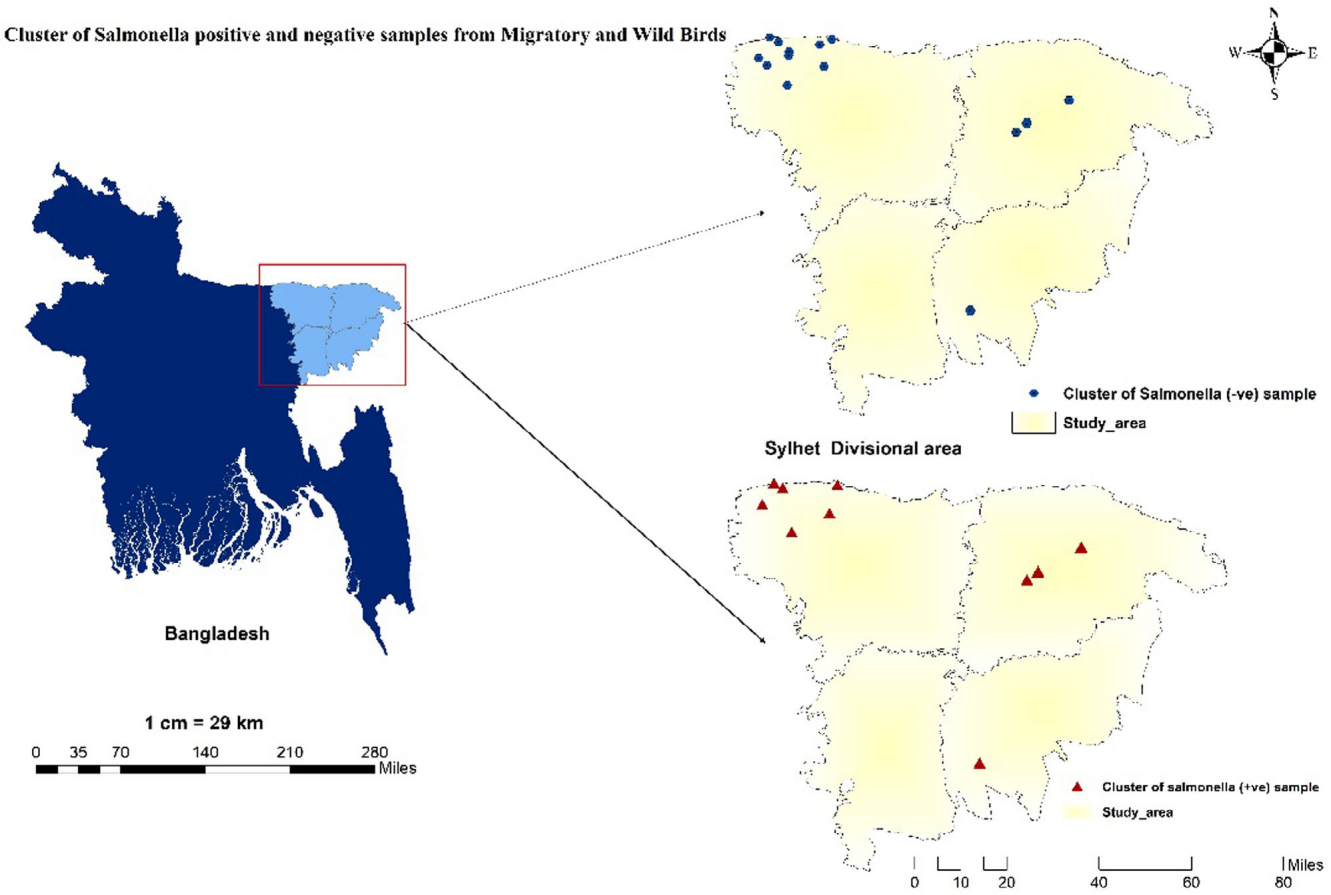
Map showing the cluster of negative and positive *Salmonella* in study area.

The Spearman correlation coefficient (*r* ^2^) was calculated using the ‘cor’ function, and its significance was assessed using the ‘cor.test’ function. The resulting coefficient values were then visualised using the ‘corrplot’ package in R and RStudio (Version 4.2.1). To visualise geospatial data, such as latitude and longitude coordinates of sample clusters, an *XY*‐coordinate map was created, where the latitude values were assigned to the *X*‐axis and the longitude values to the *Y*‐axis. Finally, the map was constructed using ArcMap 10.7 (Figure 1).

## 3 Results

From captive wild and migrating birds, a total of 262 samples were obtained. There was an estimated 30.92% (81/262) overall prevalence of *Salmonella* spp. (95% CI = 25.63–36.75). However, migrating birds had the highest incidence (38.10%) compared to wild birds kept in captivity (23.40%) (Table 2).

**TABLE 2:** Univariate association between PCR positives *Salmonella* with different category of birds.

Variable (factors)	Category	*n*/*N*	% of *n* (95% CI)	χ^2^ or Fisher exact test	*p* value
Type of birds				6.55	0.01
	Migratory	51/134	38.1% (29.82–46.84)		
	Wild	30/128	23.4% (16.41–31.37)		
	Total	81/262	30.92% (25.37–36.90)		
Location				27.13	<0.001
	Bondor	6/15	40.0% (16.34–67.71)		
	Eco park	18/57	31.6% (19.91–45.24)		
	Haripur	8/17	47.1% (22.98–72.19)		
	Sunamganj	37/94	39.4% (29.44–49.98)		
	Zoo	12/71	16.9% (9.05–27.66)		
Species of birds				35.35^b^	0.006^b^
	Black crowned night heron	1/6	16.7% (0.42–64.12)		
	Dove	0/4	N/A		
	Eagle	0/6	N/A		
	Golden pigeon	1/2	50.0% (1.26–98.74)		
	Gray parrot	5/8	62.5% (24.49–91.48)		
	Gray heron	2/8	25.0% (3.19–65.09)		
	Hornbill	0/5	N/A		
	Kalim	0/11	N/A		
	Kite	1/3	33.3% (0.84–90.57)		
	Macaw	1/3	33.3% (0.84–90.57)		
	Mathura	0/3	N/A		
	Moyna	0/8	N/A		
	Parrot	3/8	37.5% (8.52–75.51)		
	Peacock	2/10	20.0% (2.52–55.61)		
	Purple heron	0/3	N/A		
	Red jungle fowl	0/4	N/A		
	Silver pigeon	0/2	N/A		
	Sun conure	1/5	20.0% (0.51–71.64)		
	Vulture	3/3	100.0% (29.24–100.00)^a^		
	Wild (unidentified)	10/26	38.5% (20.23–59.43)		

Abbreviations: CI = confidence interval, *n* = positive case, *N* = total sample, N/A = not applicable.

^a^ One‐sided 97.5% confidence interval.

^b^ Fisher exact test value (more than 20% cells have expected count less than 5).

### 3.1 Prevalence of *Salmonella* in Different Locations

Haripur had the highest prevalence (47.1% [22.98–72.19]), whereas the zoo had the lowest (16.9% [9.05–27.66]). The prevalence rates of Bondor Bazaar, Sunamganj and Eco Park were found to be 40.0% (16.34–67.71), 39.4% (29.44–49.98) and 31.6% (19.91–45.24), respectively (Table 2). However, the differences were statistically significant (*p* < 0.001) (Table 2).

## 4 Prevalence of *Salmonella* in Different Species of Captive Wild Birds

According to this study, among the captive wild birds, vultures exhibited the highest (100%) prevalence of *Salmonella* spp., whereas the lowest prevalence was reported in black‐crowned night herons at 16.67%. However, parrots, macaws and kites exhibited a prevalence of more than 30%. *Salmonella* spp. was found to be 20% more common in sun conures and peacocks. However, the incidence of one unidentifiable wild captive bird was 38.5% (Table 2). The association between *Salmonella* and wild birds was statistically significant (*p* = 0.006) (Table 2)

### 4.1 Occurrence of Virulence Genes

The *invA* gene was found to be positive in all tested isolates. Out of the tested isolates, the virulence genes *SopE* and *agfA* were 20.99% and 24.69%, respectively (Table 3).

**TABLE 3:** Overall occurrence of the virulence genes in *Salmonella* positive samples.

Virulence gene	Occurrence (%)	95% CI
*AgfA*	20 (24.69%)	15.78–35.53
*sopE*	17 (20.99%)	12.73–31.46
*invA*	81 (100%)	95.55–100.00^a^

Abbreviation: CI = confidence interval (%).

^a^ One sided 97.5% confidence interval.

### 4.2 Antimicrobial Susceptibility Test

**FIGURE 2:**
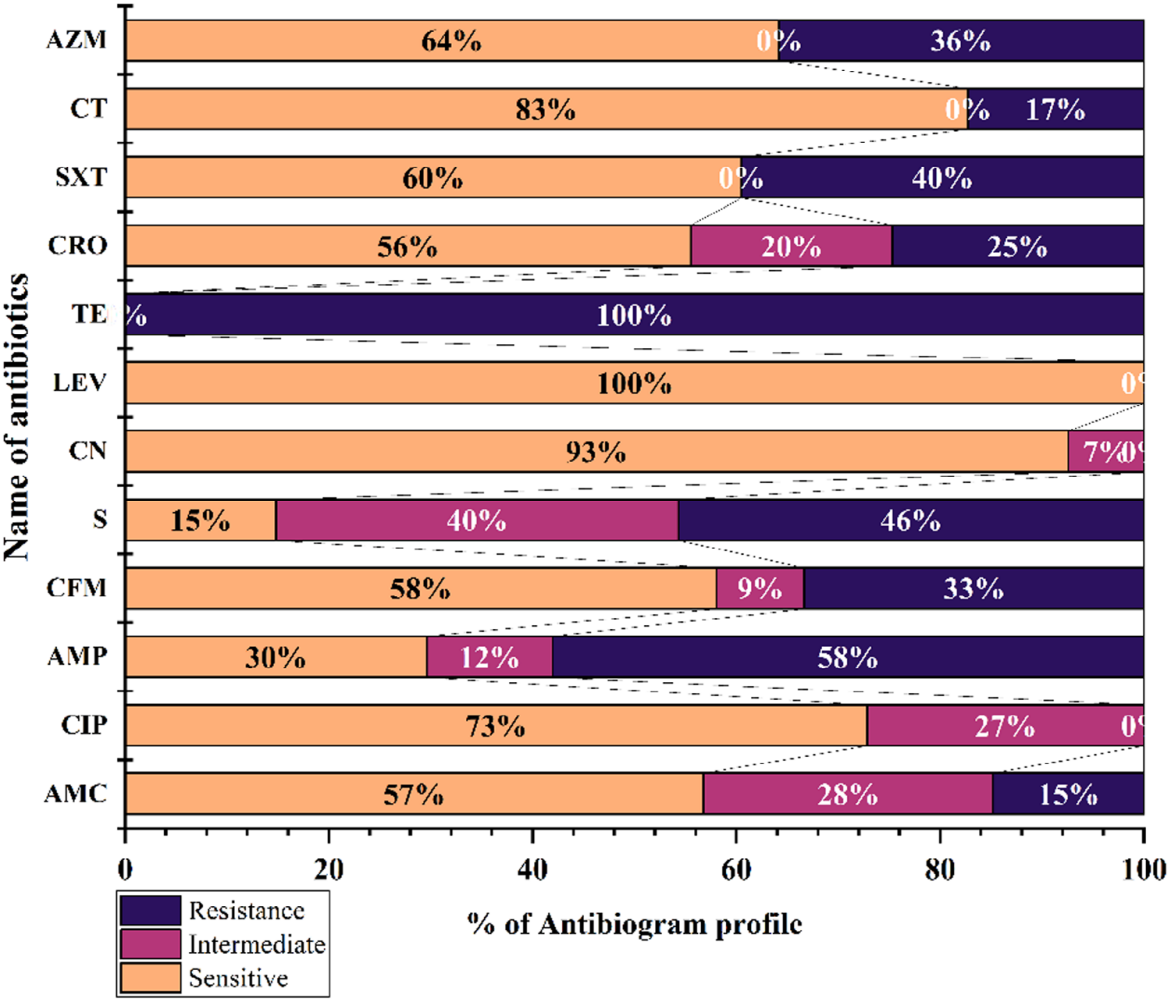
Antibiotic resistance profile of isolated *Salmonella* spp.

A total of 81 isolates from 8 groups were examined with 12 different antibiotics. The most notable resistance, at a rate of 100%, was observed in the case of tetracycline (TE) followed by 58% and 46% resistance to ampicillin (AMP) and streptomycin (S), respectively. In addition, there was a resistance rate of 17% for colistin sulphate (CT) and 25% for ceftriaxone. Interestingly, levofloxacin alone shows 100% sensitivity in all the isolates, whereas ciprofloxacin and azithromycin showed 73% and 64% sensitivity, respectively (Figure 2).

**FIGURE 3:**
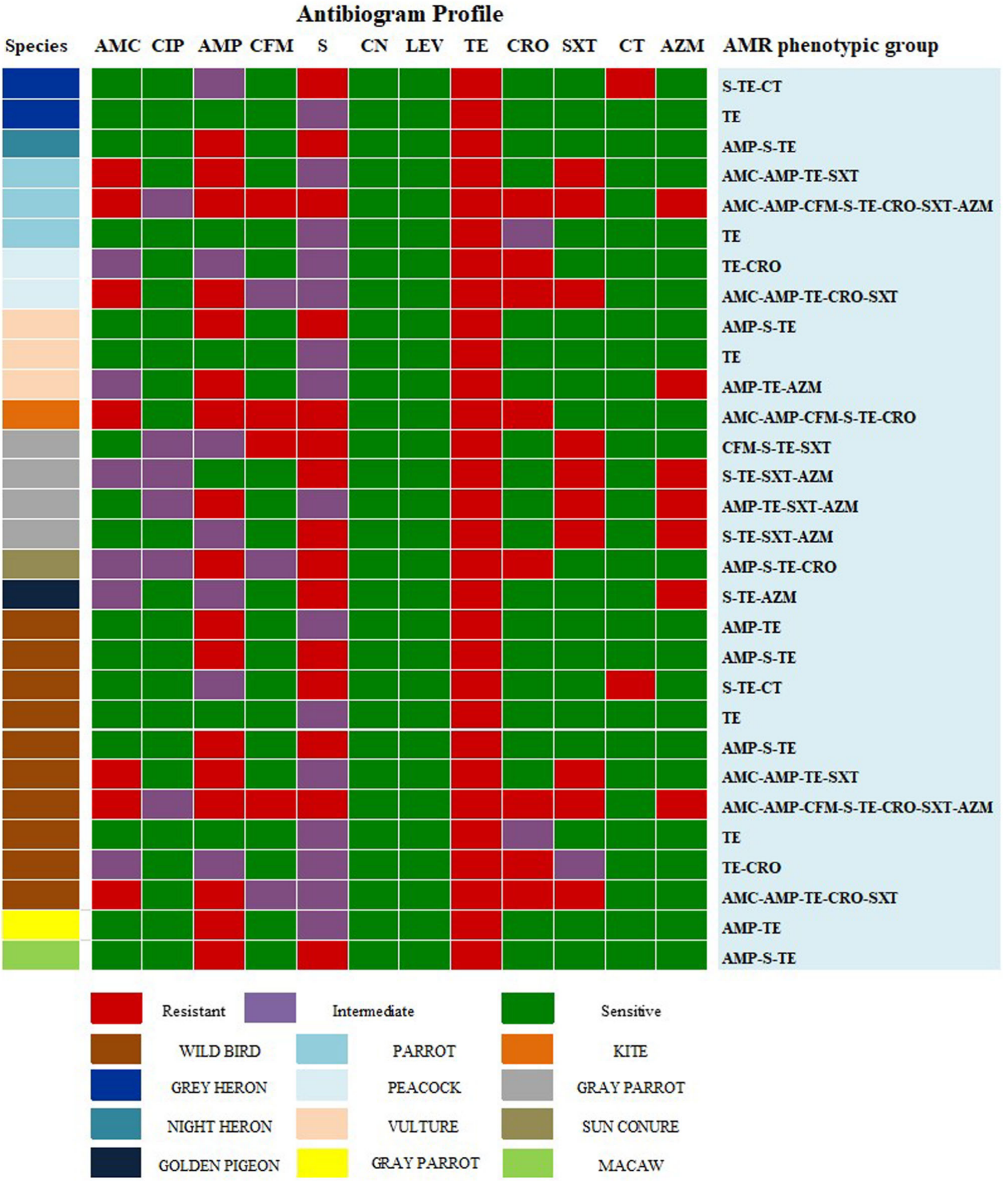
Antibiogram profile for captive wild birds.

**FIGURE 4:**
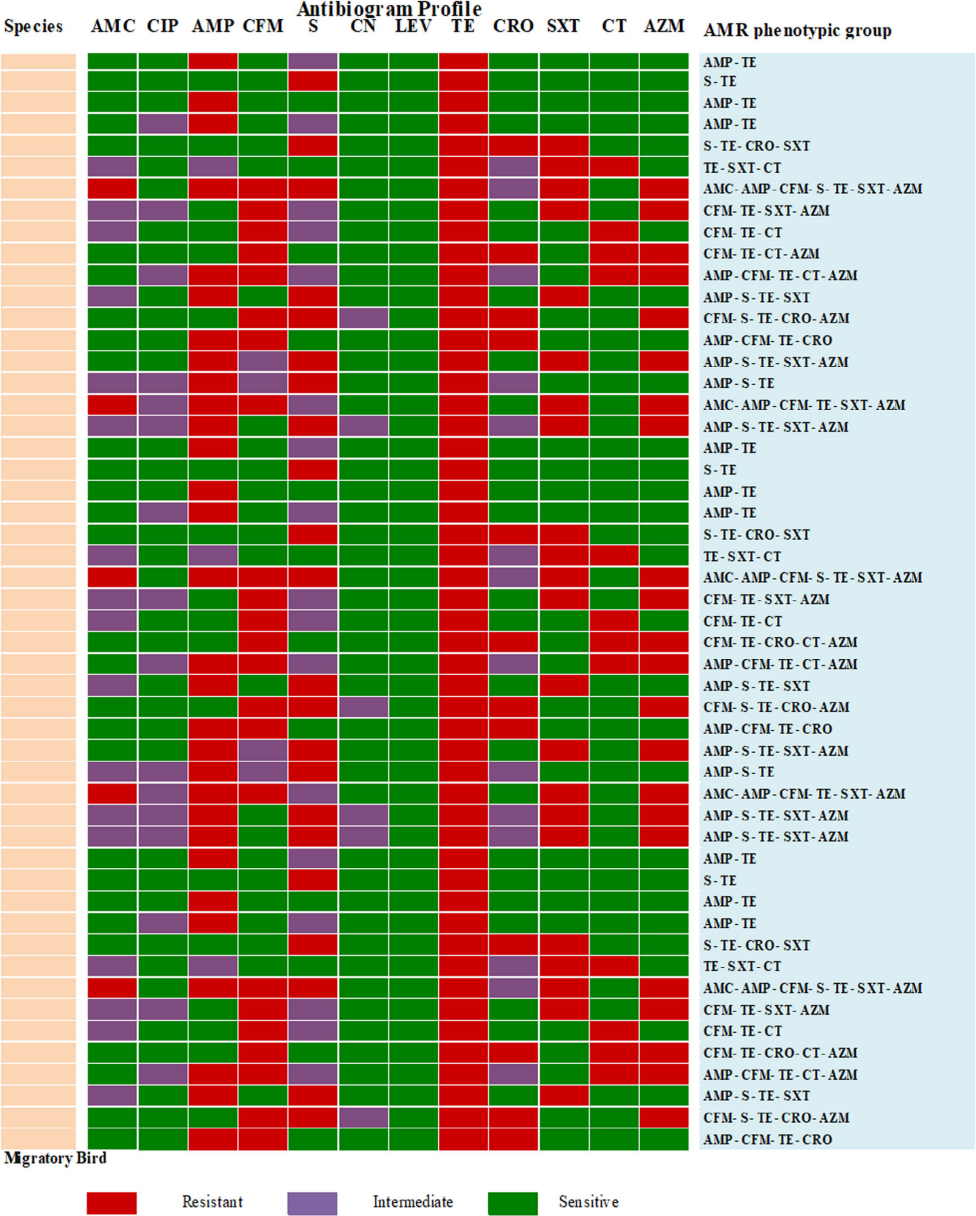
Antibiogram profile for migratory birds.

The data are shown in Table 4. *Salmonella* spp. had the highest MAR index value of 0.67 with the most often observed values of 0.25 and 0.42. Among all isolates, 74.07% (60/81) had MDR, with the ampicillin–streptomycin–tetracycline being the most prevalent pattern. However, wild bird isolates exhibited maximum resistance against eight antibiotics (AMC–AMP–CFM–S–TE–CRO–SXT–AZM) (Figure 3), whereas migratory bird isolates displayed maximum resistance against seven antibiotics (AMC–AMP–CFM–S–TE–SXT–AZM) (Figure 4).

**TABLE 4:** Antimicrobial resistance patterns of isolated *Salmonella* spp. from migratory and captive wild birds.

Pattern no.	Antibiotic resistance patterns (ARP)	No. of resistance antibiotics	Total no. of isolates	Overall MDR isolates (%)	MAR index
01	AMP‐TE	2	11	60 (74.07%)	0.17
02	S‐TE	2	03		0.17
03	S‐TE‐CRO‐SXT	4	03		0.33
04	TE‐SXT‐CT	3	03		0.25
05	AMC‐AMP‐CFM‐S‐TE‐SXT‐AZM	7	03		0.58
06	CFM‐TE‐SXT‐AZM	4	03		0.33
07	CFM‐TE‐CT	3	03		0.25
08	AMC‐AMP‐CFM‐S‐TE‐CRO‐SXT‐AZM	8	02		0.67
09	AMC‐AMP‐CFM‐TE‐SXT‐AZM	6	02		0.50
10	AMC‐AMP‐CFM‐S‐TE‐CRO	6	01		0.50
11	AMP‐CFM‐TE‐CT‐AZM	5	03		0.42
12	AMP‐S‐TE‐SXT‐AZM	5	05		0.42
13	CFM‐S‐TE‐CRO‐AZM	5	03		0.42
14	CFM‐TE‐CRO‐CT‐AZM	5	02		0.42
15	AMC‐AMP‐TE‐CRO‐SXT	5	02		0.42
16	AMP‐TE‐SXT‐AZM	4	01		0.33
17	AMC‐AMP‐TE‐SXT	4	02		0.33
18	AMP‐CFM‐TE‐CRO	4	03		0.33
19	CFM‐TE‐CT‐AZM	4	01		0.33
20	AMP‐S‐TE‐SXT	4	03		0.33
21	AMP‐S‐TE‐CRO	4	01		0.33
22	S‐TE‐SXT‐AZM	4	02		0.33
23	CFM‐S‐TE‐SXT	4	01		0.33
24	AMP‐S‐TE	3	07		0.25
25	AMP‐TE‐AZM	3	01		0.25
26	S‐TE‐AZM	3	01		0.25
27	S‐TE‐CT	3	02		0.25
28	TE‐CRO	2	02		0.17
29	TE	1	05		0.08
	Total no. isolates		81 (100%)		

**FIGURE 5:**
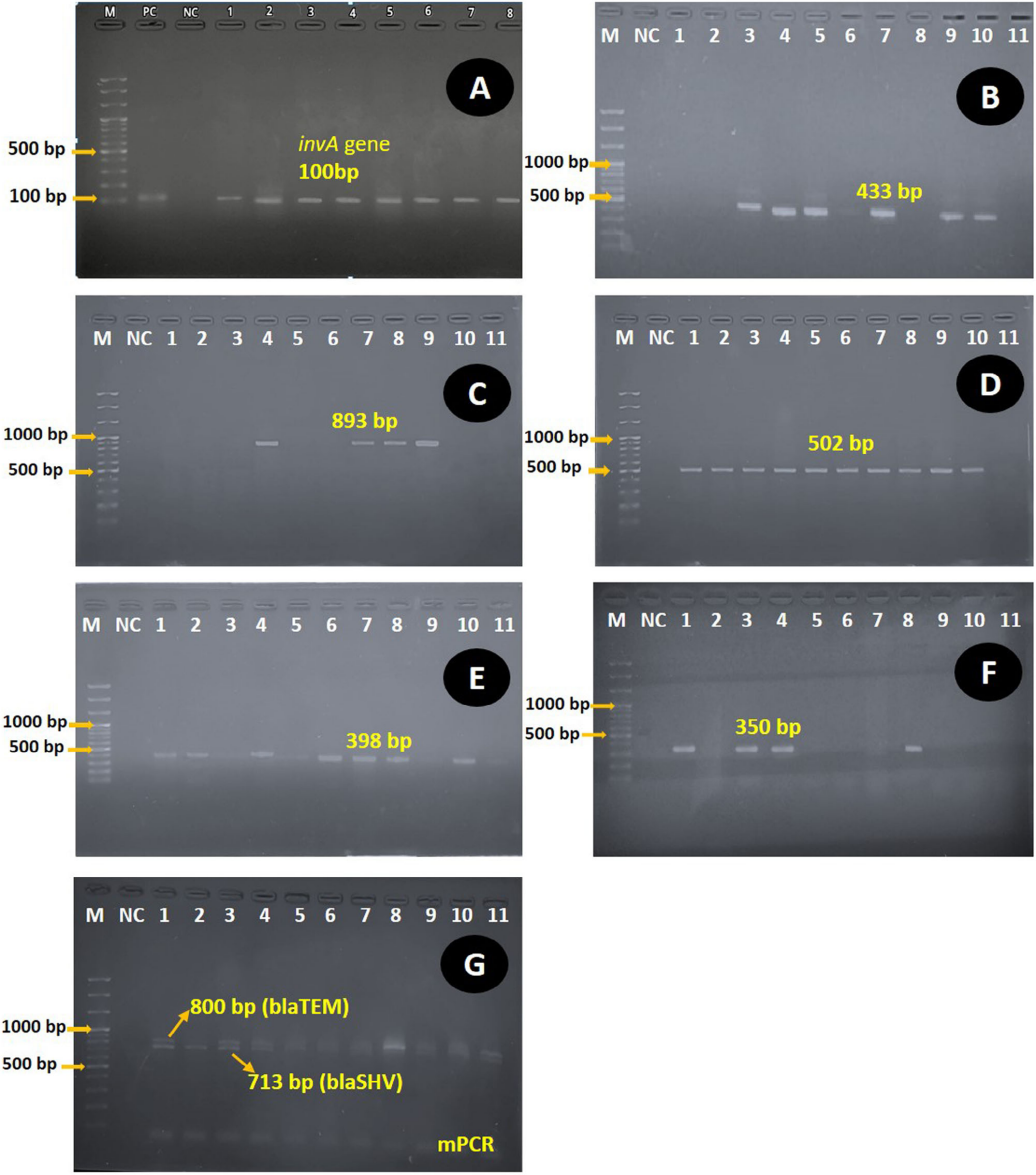
(A–G): Electrophoresis on 1.5% agarose gel, showing specific amplified band of different genes of *Salmonella* spp. amplification by PCR; Lane M: 100 bp marker DNA; Lane NC: control (–ve); A: Lane (1–8) reaction specific (+ve) for *invA* gene (100 bp); B: Lane (1–11) samples for *sul1* gene (433 bp); C: Lane 1–11 samples for *strA* gene (893 bp); D: Lane 1–11 for *tetA* gene (502 bp); E: Lane (1–11) samples *sopE* gene; F: Lane (1–11) samples for *agf*A gene (350 bp); G: multiplex PCR for ESBL producing *blaTEM* (800 bp) and *blaSHV* (713 bp).

**FIGURE 6:**
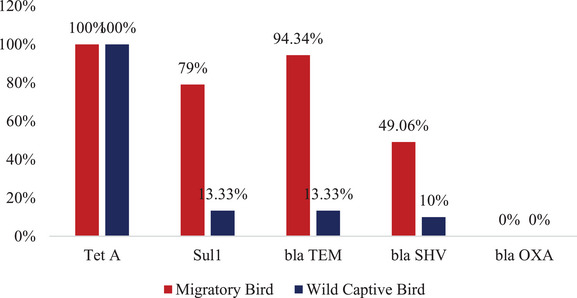
The percentage of AMR genes detected from *Salmonella* positive isolates.

Both migratory and captive wild birds were found to carry the AMR genes *blaTEM* (94.34% and 49.06%) and *blaSHV* (13.33% and 10%), respectively (Figure 5). Nevertheless, neither of the two types of bird *Salmonella* isolates had the *blaOXA* gene. Both the migratory and wild birds had sulphonamide (*sul1*) resistance genes 79% and 13.33%, respectively. Both cases showed 100% resistance to tetracycline (Tet A) (Figure 6).

**FIGURE 7:**
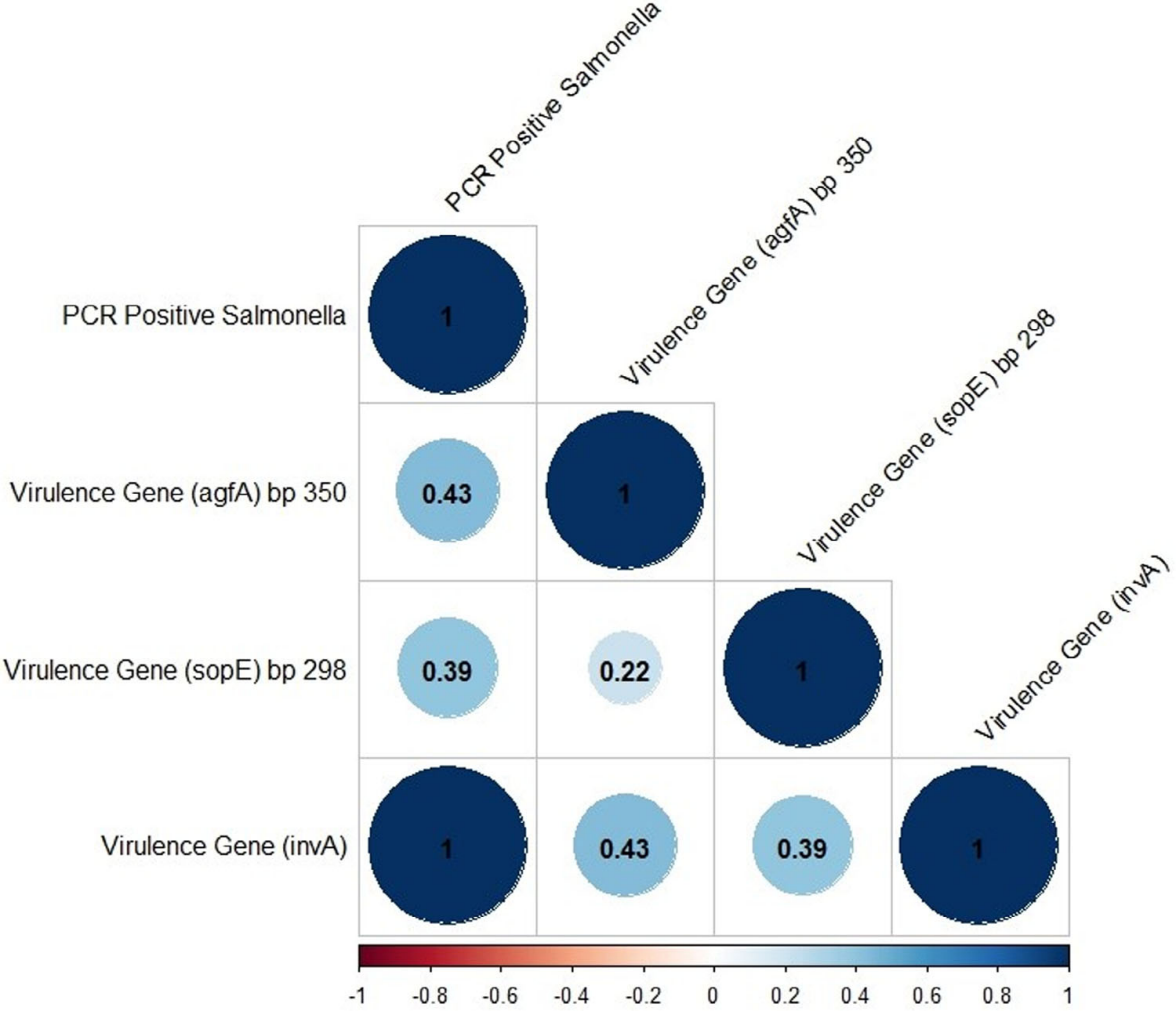
Spearman's correlation among different virulent genes and *Salmonella* positive samples.

The correlation coefficients (*r*) among different virulent genes and *Salmonella* positive samples (PCR results) are displayed in Figure 7. Blue colour represents the positive correlations. The intensity of the colour reflects the strength of the correlation. The *r* value ranging from 0 to 1 indicates positive correlation and from 0 to −1 indicates negative correlation. The figure exhibits only monotonic positive correlation and is statistically significant at the *p* = 0.01 level (two‐tailed) among different virulent genes. The coefficient (*r* = 1, *p* = 0.01) of *invA* shows a strong positive correlation with PCR positive samples, whereas others showed moderately strong correlation.

## 5 Discussion

This cross‐sectional bacteriological study was conducted to isolate and identify *Salmonella* from migratory and captive wild birds. According to Tsiodras et al. (2008), the gut microbiota contain *Salmonella* spp., which are widely present in nature. Many wild bird species have been reported to die from salmonellosis, yet these bacteria have also been discovered in birds that appear to be in good health (Najdenski et al. 2018). It is important to highlight that salmonellosis is one of the known causes of mortality in migratory birds.

The results of the current study indicate that the overall isolation rate of *Salmonella*, based on culture and PCR tests, was 30.92%. Notably, migratory birds accounted for 38.10%, whereas captive wild birds contributed 23.40%. This finding is comparable to a previous study (Saiful Islam et al. 2021) wherein it was established that 21.21% of *Salmonella* cases were attributed to migratory birds. However, another study suggested 13.4% *Salmonella* cases in migratory birds, which is much lower than our findings (Card et al. 2023). Studies from other nations revealed overall *Salmonella* prevalence, such as South Korea (0.93%), Singapore (0.99%), Uganda (4.3%), Poland (6.4%), Southern Europe (20.8%) (Afema and Sischo 2016; Antilles et al. 2021; Card et al. 2023; Krawiec et al. 2015) and Czech Republic (24%) (Dolejská et al. 2009). Conversely, compared to our study of captive wild birds, 46.67% of *Salmonella* was found in captive wild birds from the Dhaka Zoo (Al Faruq et al. 2016). *Salmonella* prevalence was found to be 67% in Asian pied starlings and 65% in Corvus splendens, or house crows (Al Faruq et al. 2016). Surprisingly, this phenomenon surpasses our empirical findings of avian species in their natural habitat. This might be the case given that starlings and house crows frequently eat carrion and get their food from waste in metropolitan areas. In the case of migrating birds, samples were collected from Sunamganj (Tanguar Haors), Bondor Bazaar and Haripur, where prevalence rates were 39.1%, 40% and 47.1%, respectively. Similar results from a previous study (Card et al. 2023) yielded comparable findings, revealing a prevalence of 16% in Hakaluki Haors and 41% in Tanguar Haors. The prevalence percentage of *Salmonella* in wild birds recorded in the zoo is 16.9% and in the Eco Park is 31.6%. These differences may be due to Eco Park being nearly free to human access or visitors compared to zoos where public entry is restricted. As we considered species variation in wild bird sampling, the highest percentage was recorded in vultures (100%), parrots, macaws and kites (above 30%) and black‐crowned night herons (16.67%). The identification of *Salmonella* in wild bird species is hindered by a lack of available data. However, Dhaka Zoo has discovered that 33.33% *Salmonella* can be found in both the Alexandrine parakeet and rose‐ringed parakeet (Al Faruq et al. 2016).

According to earlier discoveries from global studies, each isolate of *Salmonella* spp. possesses the virulence genes *invA, agfA* and *sopE*. The *sopE* gene, which is conserved in *Salmonella* pathogenic strains, is detected in 20.99% of cases. Similarly, the *agfA* gene, which is involved in biofilm formation and adhesion during infection, is found in 24.69% of the isolates. Regarding the virulence genes in migratory and wild birds, there is a lack of knowledge. In line with the previous study, we found that *Salmonella* exhibited resistance to tetracycline (100%), ampicillin (58%), streptomycin (46%), azithromycin (36%), ceftriaxone, a third‐generation cephalosporin (25%) and colistin sulphate (17%). Interestingly, levofloxacin demonstrated complete sensitivity. More prevalent patterns are ampicillin–streptomycin–tetracycline, while the occurrence of MDR is 74.07%. The development of antibiotic resistance in these bacteria could be the result of several factors, including strong selective pressure resulting from the indiscriminate use of antibiotics (Muteeb et al. 2023). In migratory birds, the utmost resistance is shown against seven antibiotic agents (AMC–AMP–CFM–S–TE–SXT–AZM), while captive wild birds displayed resistance against eight antibiotic agents (AMC–AMP–CFM–S–TE–CRO–SXT–AZM). These findings closely resembled the outcomes of previous research, which found that 11.54% of *Salmonella* isolates were ceftriaxone resistant, a third‐generation cephalosporin drug used to treat severe *Salmonella* infections in humans (Saiful Islam et al. 2021). Another study demonstrated that fluoroquinolone drugs, such as ciprofloxacin, showed sensitivity against *Salmonella*, which is consistent with the findings of our investigation.

In this study, the resistance gene was studied, and emphasis was given to the extended spectrum β‐lactamase (ESBL) gene, along with other available genes in our laboratory. The present study reported the detection of *tetA* (100%) in both migratory and captive wild birds. This is comparable to phenotypic findings, where tetracycline showed 100% resistance. The presence of *sul1* was found to be 79% and 13.33% in migratory and captive wild birds, respectively, whereas phenotypically it was found to be 43.14% in migratory birds and 33.33% in captive birds. The prevalence of *blaTEM* was the highest in migratory birds at 94.34%, while *blaSHV* was the lowest in wild birds (10%). Phenotypic and genotypic results may differ due to human errors and test sensitivity. However, the presence of numerous resistance genes ensures the occurrence of genotypic resistance (Shivakumaraswamy et al. 2019). These genetic elements may potentially promote the emergence of multidrug‐resistant pathogens. The revealed antibacterial profiles indicate that various classes of antibiotics have been utilised inappropriately and excessively, neglecting the detrimental consequences of AMR. Additionally, cross‐contamination makes it easier for bacteria to develop resistance to a variety of antibiotics in various environmental elements. Owing to their frequent resistance to treatment, multidrug‐resistant bacteria pose a serious threat to public health. According to the findings of our study, migrating birds may spread MDR bacteria from their habitats to distant areas where there is no history of widespread public awareness of how to avoid and control infectious diseases. Furthermore, migratory birds can transport and transmit MDR bacteria over great distances if they carry them (Stępień‐Pyśniak et al. 2018).

Though the study findings are interesting, this study has a few limitations. First, faecal samples were collected soon after defecation from captive wild birds. Despite taking precautions, there may be a possibility of environmental contamination. Second, the disk diffusion method is no longer recommended for colistin due to its interaction with the components of Mueller–Hinton agar, which can lead to unreliable results. Third, since the study was carried out in a specific region, the results may not fully represent the prevalence of *Salmonella* in other areas of Bangladesh. Finally, due to resource constraints, not all AMR genes were analysed, limiting the full genetic characterisation of resistant strains in the study.

## 6 Conclusion

This study underscores the significant prevalence of *Salmonella* spp. and its AMR patterns in migratory and captive wild birds in the Sylhet region of Bangladesh. The notably higher prevalence in migratory birds, alongside the presence of MDR and virulence genes, raises serious public health concerns. The detection of AMR genes such as *blaTEM* and *sul1* further highlights the potential risk of spreading resistant pathogens. Continued monitoring, along with a One Health approach, is crucial for managing the threat these resistant strains pose to both human and animal health.

## Author Contributions

**Ruhena Begum**: data curation, formal analysis, writing–original draft, writing–review and editing, methodology. **Nilima Akther Asha**: data curation, methodology, formal analysis, writing–original draft, writing–review and editing. **Diponkar Chandra Chanda Dipu**: methodology, data curation, formal analysis, writing–original draft, writing–review and editing. **Milton Roy**: formal analysis, writing–original draft, writing–review and editing. **Asikur Rahman**: formal analysis, writing–original draft, writing–review and editing. **Md. Shahidur Rahman Chowdhury**: formal analysis, writing–original draft, writing–review and editing. **Hemayet Hossain**: software, formal analysis, writing–original draft, writing–review and editing. **Md. Rafiqul Islam**: methodology, formal analysis, supervision, writing–original draft, writing–review and editing. **Md Bashir Uddin**: methodology, writing–original draft, writing–review and editing, formal analysis. **Md. Mahfujur Rahman**: methodology, writing–original draft, writing–review and editing, formal analysis. **Md. Mukter Hossain**: conceptualization, methodology, investigation, formal analysis, supervision, funding acquisition, project administration, writing–original draft, writing–review and editing.

## Ethics Statement

The authors have nothing to report.

## Conflicts of Interest

The authors declare no conflicts of interest.

### Peer Review

The peer review history for this article is available at https://www.webofscience.com/api/gateway/wos/peer-review/10.1002/vms3.70102.

## Data Availability

All data generated and analysed in this study are included in the main manuscript.
